# Human atrial extracellular vesicles suppress NLRP3 inflammasome activation and profibrotic signaling in a patient-specific iPSC model of postoperative atrial fibrillation

**DOI:** 10.7150/thno.127433

**Published:** 2026-03-30

**Authors:** Noreen Ahmed, Bin Ye, Wenbin Liang, Buu-Khanh Lam, Fraser Rubens, Saad Khan, David Courtman, Duncan John Stewart, Darryl Raymond Davis

**Affiliations:** 1 University of Ottawa Heart Institute, Division of Cardiology, Department of Medicine, University of Ottawa, Ottawa, Canada K1Y4W7.; 2 Department of Cellular and Molecular Medicine, University of Ottawa, Ottawa, Canada K1H8M5.; 3 Rhythm Biotherapeutics Inc, Ottawa, Canada K1Y4W7.; 4 University of Ottawa Heart Institute, Division of Cardiac Surgery, Department of Surgery, University of Ottawa, Ottawa, Canada K1Y4W7.; 5 Ottawa Hospital Research Institute, Division of Regenerative Medicine, Department of Medicine, University of Ottawa; Ottawa, Canada.

**Keywords:** postoperative atrial fibrillation, extracellular vesicles, NLRP3 inflammasome, induced pluripotent stem cells, cardiac fibrosis

## Abstract

**Background:**

Postoperative atrial fibrillation (AF) remains a common and morbid complication of cardiac surgery, driven by inflammation and fibrosis for which targeted therapies are limited. Conventional antiarrhythmic and anticoagulant strategies have little impact on its incidence or recurrence. We previously demonstrated that a single intracardiac injection of extracellular vesicles (EVs) derived from human heart explant-derived cells prevents postoperative AF in preclinical models. The present study aimed to elucidate the mechanisms underlying this protective effect in human cells.

**Methods:**

Induced pluripotent stem cells (iPSCs) were generated from circulating mononuclear cells obtained from cardiac surgery patients at high and low risk for postoperative AF, then differentiated into atrial fibroblasts. These cells were compared with primary human atrial fibroblasts isolated from surgical tissue. Clinical-grade cardiac EVs were manufactured from heart explant-derived cells and characterized for size, surface markers, and microRNA cargo. Both iPSC-derived and primary fibroblasts were exposed to inflammatory (IL-6, TGF-β1, lipopolysaccharide) and fibrotic stimuli, with or without EV treatment. Inflammasome activation and cytokine secretion were assessed by transcript and protein analyses.

**Results:**

iPSC-derived and primary atrial fibroblasts exhibited comparable antigenic and functional profiles and efficiently internalized cardiac EVs. EV treatment markedly suppressed activation of the NLRP3 inflammasome following lipopolysaccharide and nigericin stimulation, resulting in reduced secretion of Caspase-1, IL-1β, and IL-18 and corresponding transcript downregulation. EVs also attenuated IL-6 and TGF-β1 induced fibroblast proliferation, confirming their anti-inflammatory and antifibrotic effects across cell sources and patient risk groups.

**Conclusions:**

This study establishes a patient-specific human cellular model of cardiac fibrosis, a key determinant of postoperative AF, and identifies heart-derived EVs as potent suppressors of inflammasome activation and profibrotic signaling. These findings provide mechanistic insight into the anti-inflammatory and antifibrotic actions of cardiac EVs and support their further development as a cell-free biologic for the prevention of postoperative AF.

## Introduction

Atrial fibrillation (AF) is the most common sustained arrhythmia, affecting nearly 1% of the global population 1, 2. Its onset reflects the convergence of ectopic electrical triggers and a vulnerable atrial substrate characterized by inflammation and fibrosis 3. Conventional therapies largely target rhythm or rate control but fail to address the upstream mechanisms that initiate and sustain AF.

Postoperative AF represents a particularly significant and clinically relevant subtype, arising from the intersection of transient surgical injury and pre-existing atrial remodeling 4. Activation of the NLRP3 inflammasome has emerged as a central driver of this process, promoting inflammation, fibroblast proliferation, and atrial fibrosis 5-7. We recently demonstrated that a single injection of extracellular vesicles (EVs) derived from clinical-grade human heart explant-derived cells prevents postoperative AF in a rat model of sterile pericarditis by suppressing NLRP3 activation and its downstream inflammatory cascade 8, 9. These data support the potential of EVs as cell-free biologics capable of modulating inflammasome-dependent remodeling.

However, it remains uncertain whether these mechanisms translate to human atrial biology. Traditional reliance on animal models has limited the fidelity of AF research, as animal electrophysiology and immune responses differ fundamentally from those in humans 10, 11. This limitation has become increasingly important in light of new U.S. regulatory initiatives. In 2024, the U.S. Food and Drug Administration released its *Roadmap to Reducing Animal Testing in Preclinical Safety Studies*, outlining a strategic plan to phase in human-based new approach methodologies such as organ-on-chip systems, in silico modeling, and advanced *in vitro* assays. The roadmap acknowledges that more than 90% of drugs shown to be safe and effective in animals fail in human trials and calls for validated, mechanistically grounded human models to improve predictive accuracy and accelerate drug development 12. Complementing this shift, the FDA Modernization Act 2.0 removed statutory requirements for animal testing in investigational new drug applications, formally authorizing human cell-based and computational alternatives.

Aligned with this regulatory and scientific transition, we developed a human cellular model of the substrate for postoperative AF using induced pluripotent stem cells (iPSCs) derived from cardiac surgery patients at high and low risk for the arrhythmia. These iPSCs were differentiated into atrial fibroblasts—key effector cells in atrial inflammation and structural remodeling 13— and compared with primary human atrial fibroblasts. Using this platform, we investigated how heart-derived EVs influence inflammasome activation and cytokine-induced proliferation in human atrial fibroblasts.

This study establishes the first patient-specific human model reproducing the substrate of postoperative atrial inflammation and demonstrates that cardiac EVs suppress NLRP3-mediated signaling and profibrotic remodeling. The findings provide mechanistic and regulatory justification for human-based, non-animal preclinical approaches, advancing the development of EV therapy as a cell-free biologic for preventing postoperative AF. Developing humanized preclinical platforms that replicate the postoperative inflammatory environment fulfills an urgent translational need. By integrating patient-specific genetics, fibroblast behavior, and EV-based modulation, this work defines a human theranostic model capable of predicting atrial vulnerability and evaluating candidate biologics before clinical translation.

## Results

### Patient recruitment and generation of iPSCs from high- and low-risk individuals

**Figure 1:**
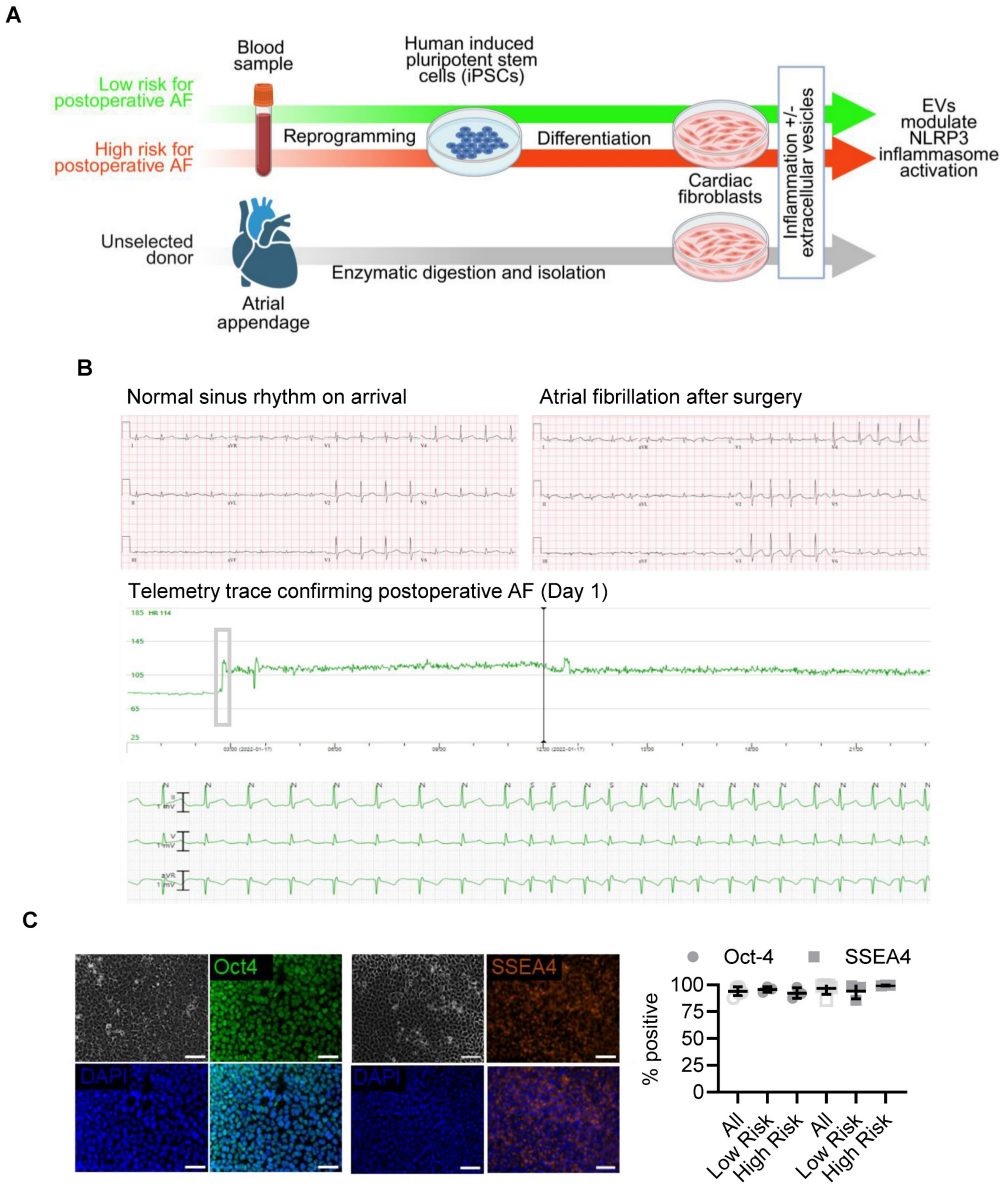
Patient-specific theranostic workflow integrating clinical risk stratification, iPSC differentiation, and extracellular vesicle testing. (A) Schematic overview of experimental design. Peripheral blood from patients stratified by validated postoperative AF propensity scores was reprogrammed to iPSCs and differentiated into atrial fibroblasts. Human heart-derived extracellular vesicles (EVs) were applied to assess modulation of NLRP3 inflammasome activation. (B) Representative clinical electrocardiograms showing sinus rhythm on intensive-care admission and onset of postoperative AF within 24 hours after surgery, with corresponding telemetry trace confirming arrhythmia onset (Day 1). (C) Immunofluorescent staining for OCT4 and SSEA4 (left) and flow-cytometric quantification (right) demonstrate robust pluripotency marker expression in reprogrammed iPSCs from all donors (mean ± SD; n = 3 per group). Scale bars = 50 µm.

Blood samples were collected from patients before cardiac surgery for the generation of iPSCs (Figure 1A). Using a validated postoperative AF propensity scoring system 14, we recruited nine patients at increased risk for AF (> 1 point; estimated postoperative AF risk ≈ 70%) and nine at low risk (0 points; estimated postoperative AF risk ≈ 17%). As summarized in Table 1, low-risk patients were younger (57 ± 10 vs. 74 ± 5 years; *p* < 0.01) and exhibited higher estimated glomerular filtration rates (109 ± 17 vs. 68 ± 29 mL/min/1.73 m²; *p* < 0.01). The main contributors to a lower risk score were younger age (0 vs. 1.9 ± 0.6 points; *p* < 0.01) and referral for valve surgery (0 vs. 0.6 ± 0.5 points; *p* < 0.01). No participants had severe chronic obstructive pulmonary disease, an eGFR < 15 mL/min/1.73 m², an intra-aortic balloon pump, left ventricular ejection fraction < 30%, or emergency surgery.

Consistent with risk allocation 15, 16, 22% of low-risk and 44% of high-risk patients developed postoperative AF (*p* = 0.69; Figure 1B), and time to onset did not differ (3.5 ± 0.7 vs. 3.8 ± 1.5 days; *p* = 0.84). Blood from three high-risk patients who developed postoperative AF and three low-risk patients who remained arrhythmia-free were reprogrammed into iPSCs using Sendai virus 17. All lines exhibited typical pluripotent morphology and nearly 100% expression of OCT4 and SSEA4 by flow cytometry at passage 30 (Figure 1C; Figure S1) 18. Reprogramming efficiency and marker expression were comparable between high- and low-risk groups.

### Human iPSC-derived and primary atrial fibroblasts exhibit comparable inflammatory responses

**Figure 2:**
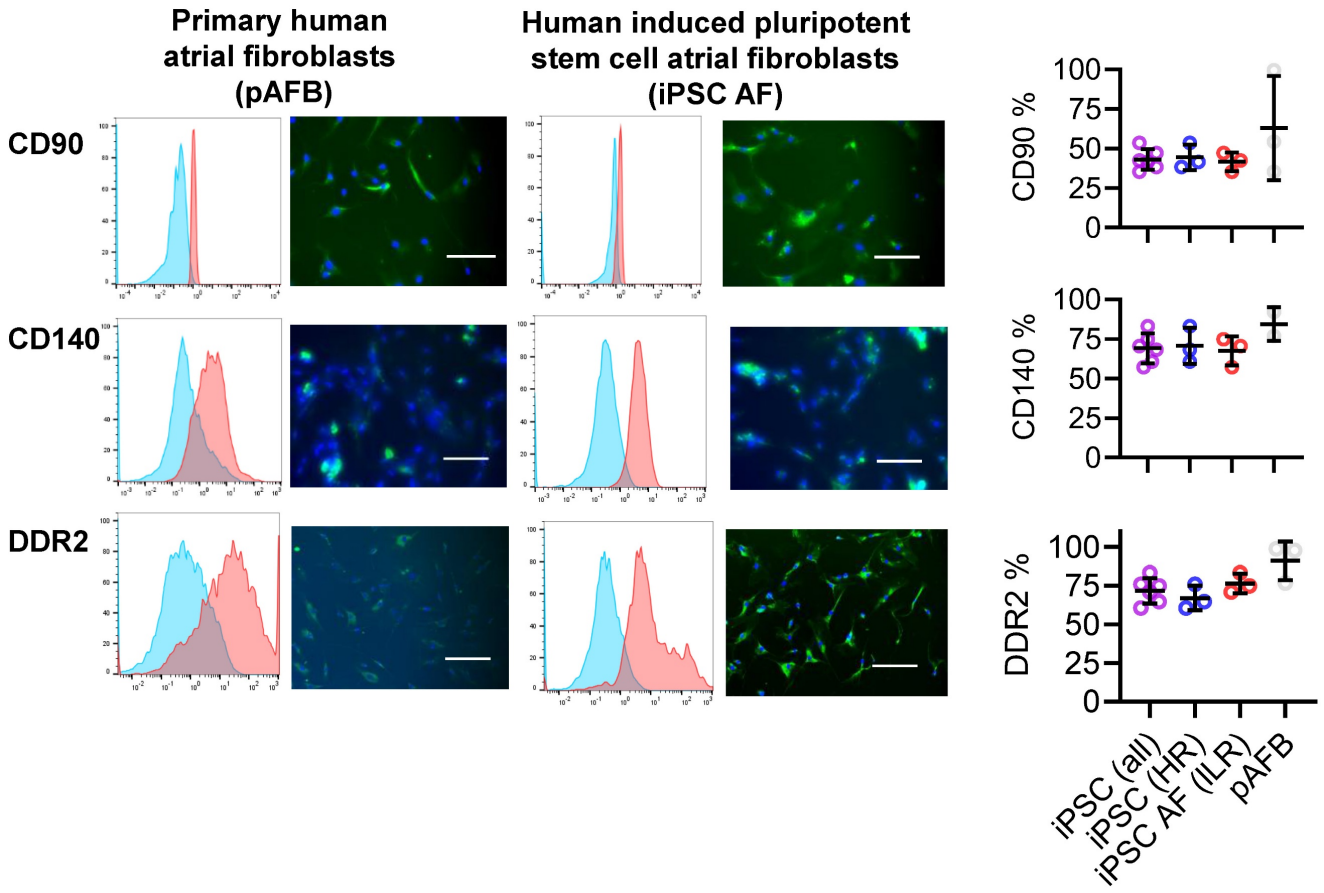
Phenotypic comparison of primary human atrial fibroblasts (pAFB) and human induced pluripotent stem cell-derived atrial fibroblasts (iPSC-AF) from patients at low and high risk of postoperative AF. Expression of fibroblast markers CD90, CD140, and DDR2 was assessed by flow cytometry (left panels) and immunofluorescence (middle panels). Quantification of flow cytometry data (right panels) demonstrates comparable marker expression in iPSC-AF from high-risk (blue) and low-risk (red) donors, as well as in pooled iPSC-AF lines (purple), relative to pAFB (grey). Immunofluorescence images show marker staining (green) and nuclear counterstaining with DAPI (blue). Data are presented as mean ± SD (n = 3 per group). Scale bars = 50 µm for CD90 and CD140, and 100 µm for DDR2.

**Figure 3:**
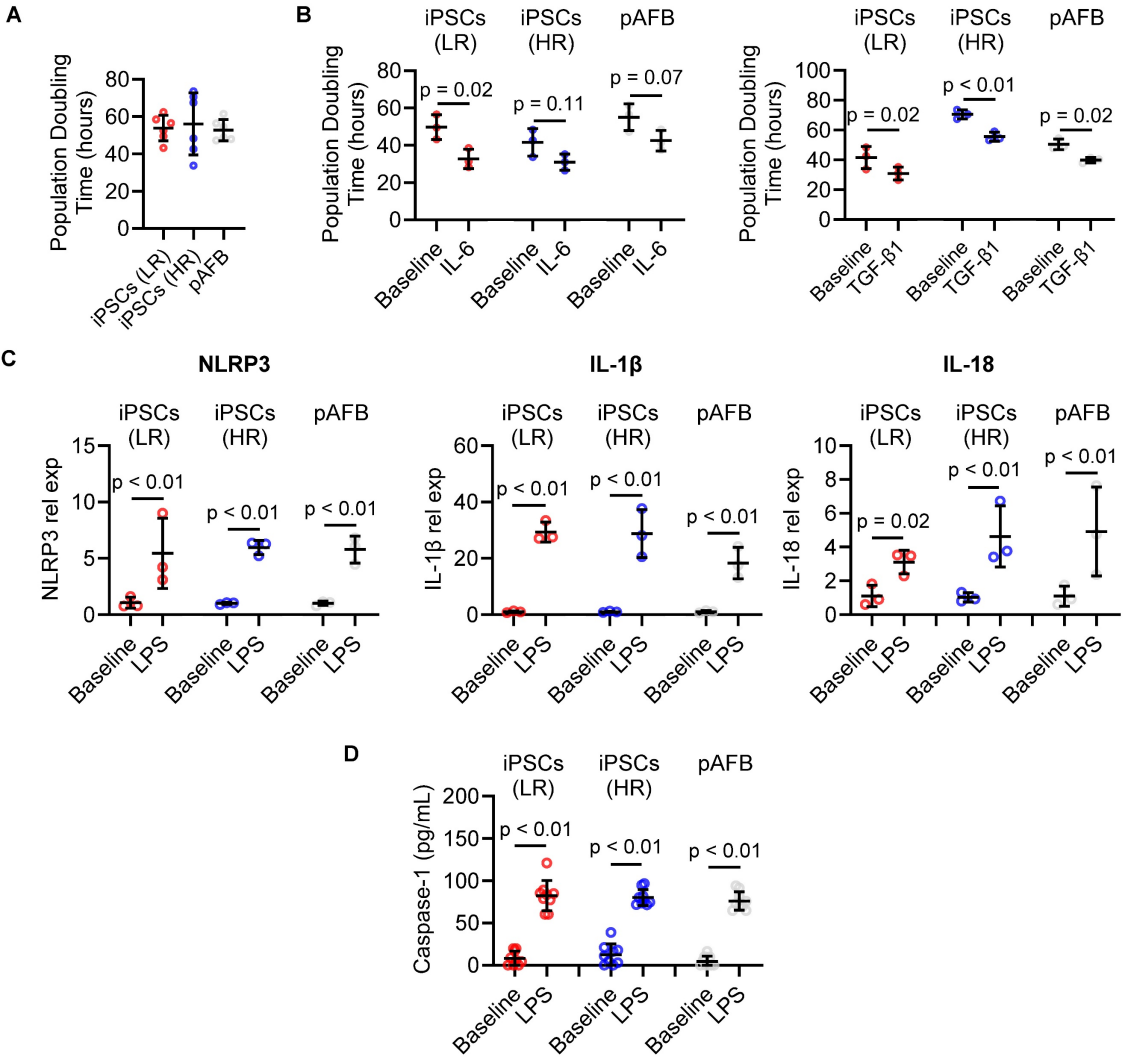
Impact of inflammatory cytokines and inflammasome activation in human atrial fibroblast models of postoperative atrial fibrillation (AF). (A) Baseline population doubling time of atrial fibroblasts derived from human induced pluripotent stem cells (iPSC-AF) from patients at low (LR, red) and high (HR, blue) risk of postoperative AF, compared with primary human atrial fibroblasts (pAFB, grey). (B) Population doubling time following exposure to interleukin-6 (IL-6, left) or transforming growth factor-β1 (TGF-β1, right) in LR and HR iPSC-AF and pAFB. (C) Relative expression of inflammasome markers NLRP3, IL1B, and IL18 at baseline and after lipopolysaccharide (LPS) stimulation in LR and HR iPSC-AF and pAFB. (D) Caspase-1 activity in LR and HR iPSC-AF and pAFB at baseline and following LPS ± nigericin stimulation. Cytokine and inflammasome activation assays demonstrate comparable inflammatory responsiveness across iPSC-derived and primary fibroblast models. Data are presented as mean ± SD; n = 3 biological replicates per group for panels A-C, and n = 9 biological replicates for panel D. Statistical comparisons were performed using unpaired Student's t-tests; p < 0.05 was considered significant (p < 0.05, p < 0.01).

Fibroblasts, which constitute approximately a quarter of atrial cells 13 and play key roles in AF pathophysiology 4, 19, were modeled using both iPSC-derived and primary human atrial fibroblasts. Primary fibroblasts were isolated from atrial appendages obtained during cardiac surgery (Table 1). iPSCs derived from 3 high and low risk patients were cultured under defined inductive conditions 20, 21 differentiated into fibroblast-like cells expressing canonical markers CD90, DDR2, and CD140a (Figure 2). Marker distribution and baseline proliferation (Figure 3A) were similar to those of primary atrial fibroblasts, confirming successful differentiation. Neither marker expression nor baseline proliferation differed between cells derived from patients with or without postoperative AF.

To model postoperative inflammatory stress, cells were exposed to IL-6 and TGF-β1, cytokines implicated in atrial remodeling 4. Both cytokines significantly accelerated proliferation by reducing population doubling time (Figure 3B). This proliferative response was consistent across iPSC-derived and primary fibroblast lines, regardless of patient risk status.

To determine whether the *in vitro* model recapitulates inflammasome activation 5, 6, 22, fibroblasts were stimulated with lipopolysaccharide (LPS). iPSC-derived fibroblasts from both risk groups upregulated *NLRP3*, *IL18*, and *IL1β* transcripts (Figure 3C), indicating intact immune responsiveness. Primary atrial fibroblasts demonstrated a similar transcriptional profile, validating the fidelity of the iPSC-derived model. No significant expression differences were detected between high- and low-risk cell lines.

Further activation with nigericin after LPS priming increased secreted Caspase-1 levels in supernatants (Figure 3D), confirming NLRP3 inflammasome activation in both iPSC-derived and primary fibroblasts. Caspase-1 induction did not differ between risk groups, reinforcing the equivalence of their inflammatory response.

### Cardiac EVs suppress inflammasome activation and fibroblast proliferation

**Figure 4:**
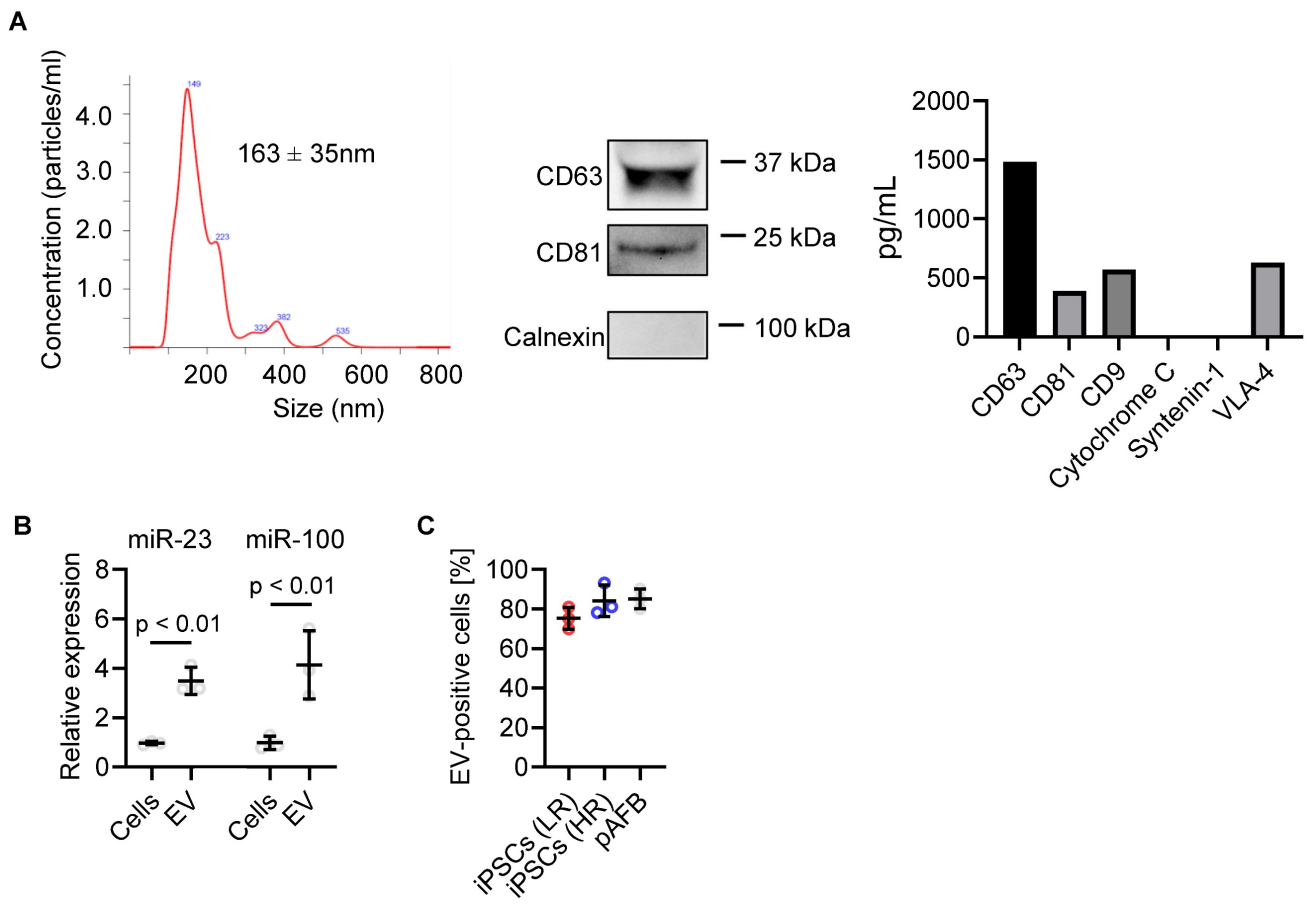
Characterization of extracellular vesicles (EVs) produced by human atrial explant-derived cells. (A) EV size distribution and concentration were determined by nanoparticle tracking analysis, showing a mean diameter of 163 ± 35 nm. Western blotting confirmed the presence of canonical EV surface markers CD63 and CD81 and the absence of the endoplasmic reticulum marker calnexin. Complementary Luminex profiling quantified both canonical and non-canonical EV proteins. (B) EV microRNA cargo was analyzed by quantitative reverse-transcription polymerase chain reaction, revealing enrichment of miR-23 and miR-100 in EVs compared with parent cells. Data represent mean ± SD (n = 3 biological replicates). Statistical comparisons were performed using unpaired Student's t-tests; p < 0.05 was considered significant. (C) Uptake of fluorescently labeled (DiD) EVs was quantified by flow cytometry following 24-hour incubation with primary atrial fibroblasts (pAFB) and induced pluripotent stem cell-derived atrial fibroblasts (iPSC-AF) from low-risk (LR) and high-risk (HR) patients, showing comparable EV internalization across all cell types.

Clinical-grade human heart-derived cells were cultured under serum-free, xenogen-free conditions within a cell manufacturing facility to produce conditioned media for EV isolation by tangential flow filtration 8. Nanoparticle tracking analysis showed a modal diameter of 163 ± 35 nm (range 95-170 nm; Figure 4A and S3). Western blotting confirmed expression of EV markers CD81 and CD63 and absence of the cellular marker Calnexin. Consistent with prior studies 7, 23, 24, EDC-derived EVs were enriched in miR-23a and miR-100a, two microRNAs known to inhibit fibroblast activation and proliferation (Figure 4B) 7, 9, 23. DiD-labeled EVs were efficiently internalized by both iPSC-derived and primary atrial fibroblasts within 24 hours (Figure 4C), independent of donor risk status.

**Figure 5:**
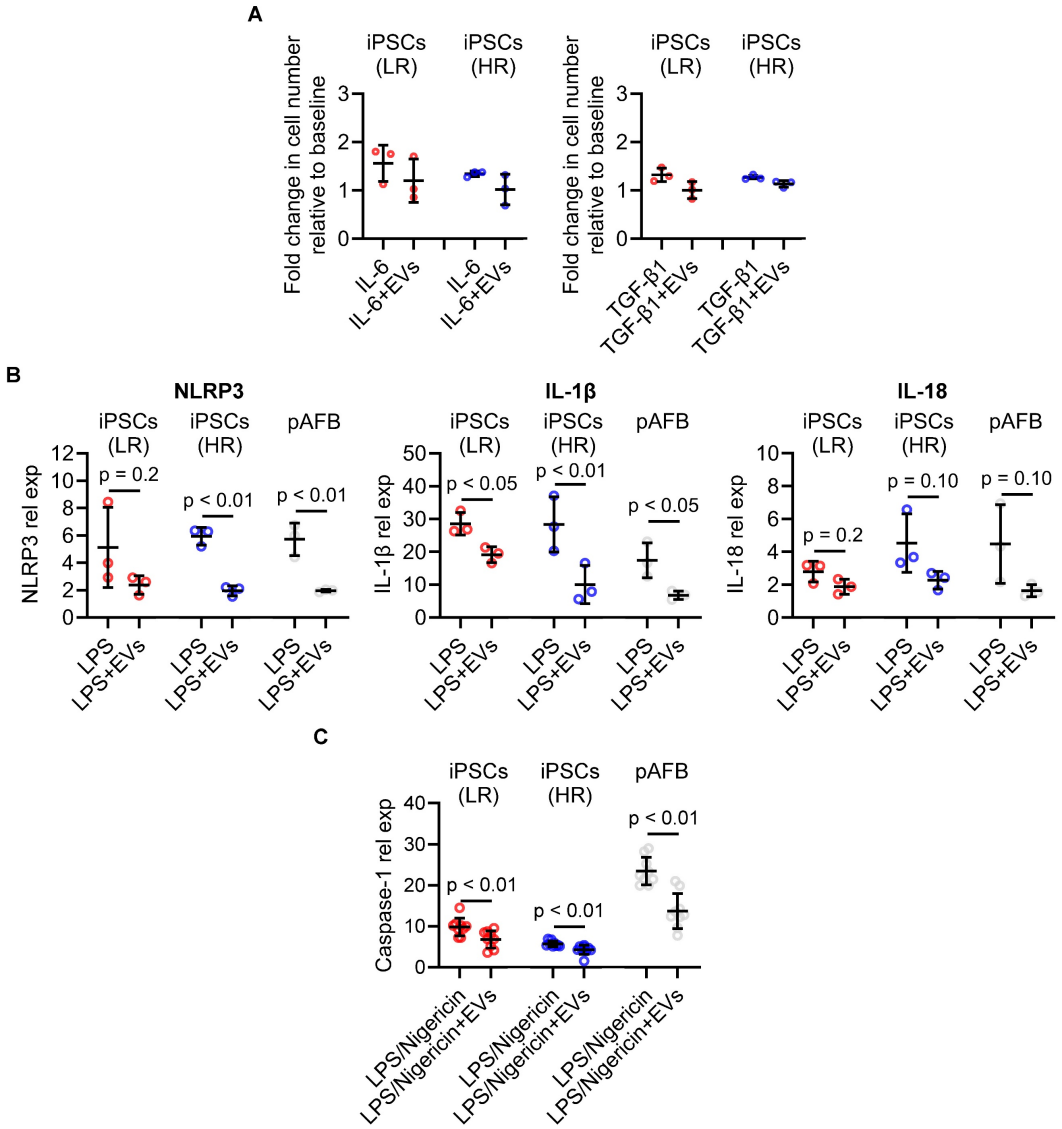
** Extracellular vesicles (EVs) suppress cytokine-induced proliferation and inflammasome activation in human atrial fibroblast models.** (A) EV treatment reduced cytokine-induced proliferation in human induced pluripotent stem cell-derived atrial fibroblasts (iPSC-AF) from low-risk (LR, red) and high-risk (HR, blue) patients, as well as in primary atrial fibroblasts (pAFB, grey). Increases in cell number induced by interleukin-6 (IL-6) or transforming growth factor-β1 (TGF-β1) were attenuated in the presence of EVs. (B) Expression of inflammasome-associated transcripts (NLRP3, IL1B, and IL18) following lipopolysaccharide (LPS) stimulation, with or without EVs, in LR and HR iPSC-AF and pAFB. EVs consistently decreased NLRP3 pathway activation and downstream cytokine gene expression. (C) Caspase-1 protein expression relative to the unstimulated baseline after sequential LPS and nigericin stimulation. Data are presented as mean ± SD (n = 3 biological replicates per group). Statistical comparisons were performed using unpaired Student's t-tests; exact p-values are indicated in each panel.

EV treatment significantly suppressed TGF-β1-induced fibroblast proliferation and showed a similar trend with IL-6 stimulation (Figure 5A). In LPS-stimulated fibroblasts, EVs markedly reduced transcript levels of NLRP3, IL1B, and IL18 (Figure 5B) and diminished protein secretion of Caspase-1 (Figure 5C). These anti-inflammatory and anti-proliferative effects were consistent across iPSC-derived and primary fibroblasts and were independent of patients' preoperative risk classification. To explore whether EVs could also attenuate an established proliferative phenotype, we performed exploratory delayed-treatment experiments in which EVs were administered after inflammatory stimulation. In this setting, EVs reduced proliferation in primary atrial fibroblasts but not in iPSC-derived fibroblasts (Figure S2).

Collectively, these data demonstrate that cardiac EVs blunt inflammasome activation and fibroblast proliferation in a patient-specific human model of postoperative atrial inflammation, providing mechanistic support for their clinical translation as a cell-free biologic therapy for the prevention of postoperative AF.

## Discussion

Postoperative AF remains a frequent and costly complication of cardiac surgery, contributing to prolonged hospital stays, greater resource use, and increased long-term morbidity 4. Current strategies, rhythm or rate control with antiarrhythmic drugs and anticoagulation, provide limited protection, often constrained by hypotension, bleeding, or proarrhythmic risks 25. Despite decades of effort, no upstream intervention has meaningfully reduced postoperative AF incidence, largely because conventional models have failed to capture the complex inflammatory and fibrotic remodeling that drives this arrhythmia in humans.

Here, we present a patient-specific, human cellular model of postoperative AF that circumvents the limitations of animal systems and directly reflects the clinical environment of cardiac surgery. By generating iPSC lines from patients stratified by validated AF propensity scores and differentiating them into atrial fibroblast phenotypes, we established a reproducible *in-vitro* platform that mirrors key molecular events observed in human atrial tissue. The comparable responses of iPSC-derived and primary atrial fibroblasts to IL-6 and TGF-β1 stimulation confirm that these cells faithfully reproduce the key inflammatory and proliferative cues implicated in postoperative atrial remodeling.

Using this system, we demonstrate that EVs released from clinical-grade human heart-derived cells exert robust anti-inflammatory and anti-fibrotic actions in human fibroblasts. EVs reduced NLRP3 inflammasome activation and downstream secretion of IL-1β, IL-18, and caspase-1, mediators long linked to atrial fibrosis and arrhythmogenic remodeling. These findings align with recent evidence identifying fibroblast-restricted NLRP3 activation as a key initiator of atrial cardiomyopathy and AF 26, and with emerging models describing bidirectional fibro-inflammatory crosstalk between fibroblasts, cardiomyocytes, and immune cells mediated by IL-6/STAT3 and miR-21 signaling 27. Moreover, suppression of inflammasome priming and triggering by cardiac EVs directly targets the atrial-specific inflammatory network termed the “atrial inflammasome,” which operates across resident cardiomyocytes and fibroblasts 28. This network is prominently up-regulated in patients who develop postoperative AF, as shown by elevated NLRP3, ASC, and gasdermin D expression in atrial tissue 5. The uniformity of response across high- and low-risk patient-derived lines suggests that EVs modulate conserved effector pathways rather than patient-specific modifiers, supporting their development as a broadly applicable, cell-free biologic for the prevention of postoperative AF. The present study was designed to establish human pathway engagement and translational fidelity, rather than to deconvolute individual EV cargo components, which will be the focus of future mechanistic studies.

While IL-1β and IL-18 are key downstream mediators of NLRP3 inflammasome activation and have been implicated in the fibro-inflammatory remodeling associated with postoperative AF, we did not use these cytokines as exogenous stimuli in the present study. Our intent was to model upstream inflammatory conditioning and inflammasome activation rather than bypass these processes by direct downstream stimulation. Accordingly, IL-6 was selected as a clinically relevant perioperative inflammatory signal that promotes fibroblast activation and proliferation, while LPS and nigericin were used to directly interrogate canonical inflammasome priming and activation. In this framework, IL-1β and IL-18 serve as mechanistic readouts of NLRP3 activity rather than initiating stimuli. Future studies incorporating IL-1β, and IL-18, driven fibro-inflammatory signaling will further expand the scope of this human model and help delineate downstream feed-forward loops relevant to clinical postoperative AF.

While postoperative AF ultimately manifests as an electrical arrhythmia, accumulating evidence indicates that its susceptibility is conditioned by upstream inflammatory and fibrotic remodeling of the atrial substrate. Accordingly, the present study was not intended to model electrophysiological disturbances or multicellular arrhythmia dynamics directly, but rather to isolate and interrogate a causally implicated human fibro-inflammatory substrate using patient-derived cells. We view this reductionist approach as complementary to, rather than a replacement for, more complex multicellular or electrophysiological systems, which can be layered onto validated human substrate models in future work.

This study aligns with current regulatory initiatives that promote the development and implementation of human-based preclinical systems capable of generating mechanistically informative data 12. These initiatives emphasize replacing or reducing animal use through new approach methodologies that better model disease-relevant biology and improve translational predictivity. The patient-specific iPSCs-derived atrial fibroblast platform developed here fulfills these objectives by reproducing the inflammatory and fibrotic mechanisms underlying postoperative AF in a fully human context. The model enables quantifiable, pathway-specific endpoints, including NLRP3 activation, cytokine release, and fibrotic gene expression, that correspond to established clinical biomarkers. By combining mechanistic fidelity with patient-derived inputs, this system provides a scientifically robust framework for assessing potency and mechanism of action in biologic development while supporting the broader regulatory movement toward more predictive, human-relevant preclinical testing.

From a translational perspective, we envision EV therapy being administered during the acute perioperative window, either intraoperatively or immediately following separation from cardiopulmonary bypass, prior to peak inflammasome activation. Although postoperative AF typically manifests 48-72 hours after surgery, accumulating evidence suggests that the arrhythmogenic substrate is initiated during the early perioperative inflammatory and thromboinflammatory phase. In this context, EVs are best conceptualized as a biologic intervention targeting early upstream “kindling” of fibro-inflammatory signaling, rather than as a late antiarrhythmic therapy. Future studies integrating coagulation-inflammatory crosstalk will further inform optimal timing and positioning of EV-based strategies in clinical translation. Together, these considerations support perioperative EV delivery as a rational strategy to modify the atrial substrate before the onset of clinically manifest postoperative AF. Accordingly, the present findings should be interpreted as evidence of modulation of fibro-inflammatory remodeling and human pathway engagement, rather than as direct prediction of clinical efficacy or arrhythmia suppression at the whole-heart level.

Several limitations warrant mention. The sample size was small, and subtle transcriptomic differences between high- and low-risk lines may emerge in larger cohorts. The focus on IL-6 and TGF-β1 provides a reductionist view of the postoperative inflammatory milieu; future studies incorporating oxidative stress and immune-stromal interactions will deepen insight. Nonetheless, the reproducibility and mechanistic coherence of this model underscore its translational value.

In summary, this work establishes the first human iPSC-derived cellular model of postoperative atrial inflammation and demonstrates that cardiac-derived EVs can directly suppress inflammasome activation and fibroblast proliferation. Beyond clarifying the mechanistic basis of postoperative AF, these findings exemplify how next-generation, human-based platforms can accelerate the translation of biological therapeutics while aligning with evolving regulatory paradigms that prioritize alternatives to animal testing.

## Methods

### Patient recruitment, iPSCs cell culture and characterization

Human iPSCs were generated from blood samples donated by patients before undergoing cardiac surgery, in accordance with a protocol approved by the University of Ottawa Heart Institute Research Ethics Board (protocol #20150313). All procedures conformed to the principles of the Declaration of Helsinki and informed consent was obtained from all participants. Using an established clinical scoring system to assess postoperative AF risk, we recruited 12 high-risk patients, who scored above 6 points (indicating a 70% risk of postoperative AF), and 12 low-risk patients, who scored 0 points (indicating a 15% risk of postoperative AF) 14. Initially, peripheral blood mononuclear cells were cryogenically stored. During the postoperative period, we monitored patients for the occurrence and outcomes of AF. Peripheral blood mononuclear cells from the first three high-risk patients who developed postoperative AF and three low-risk patients who remained arrhythmia-free were reprogrammed into iPSCs for proof-of-concept studies. These formed two groups, each consisting of three samples, and were reprogrammed using Sendai virus (A16518, Thermo Fisher Scientific) 17.

Human iPSCs were cultured on Matrigel-coated (Corning) plates and maintained in chemically defined E8 medium (Thermo Fisher Scientific). The cells were passaged at a 1:12 ratio every four days using a gentle cell dissociation reagent (Stemcell Technologies). All iPSC lines then underwent standard assays to ensure identity and quality control 9. Single-cell iPSC suspensions were stained for Oct-4 (60093AD.1, Stemcell Technologies) or SSEA-4 (60062PE.1, Stemcell Technologies) with or without permeabilization (Thermo Fisher Scientific), as appropriate. The cells were then washed and resuspended prior to analysis by flow cytometry (Guava easyCyte 8HT Flow Cytometer, Cytek Biosciences). Data were analyzed using FlowJo software (FlowJo, LLC, Data Analysis Software). Immunohistochemistry was performed on adherent cells, utilizing the same antibodies. Cells were stained with 4′,6-diamidino-2-phenylindole (DAPI, Sigma) prior to imaging.

### Differentiation of iPSCs into cardiac fibroblasts

Human iPSCs were cultured in E8 medium until they reached 80% confluency, following previously described protocols 20, 21. Differentiation was initiated by adding RPMI/B27 medium without insulin (Thermo Fisher Scientific), supplemented with 6 μM CHIR99021 (Sigma-Aldrich), and incubated for 2 days. On day 3, the medium was replaced with RPMI/B27 without insulin and 5 μM IWR-1 (Sigma-Aldrich) for an additional 2 days. Subsequently, the cells were cultured for 24 hours in RPMI/B27 without insulin. Then, cells were passaged at a 1:12 split ratio into Advanced DMEM/Glutamax medium (Thermo Fisher Scientific), supplemented with 1% fetal bovine serum (Thermo Fisher Scientific), 5 μM CHIR99021, 2 μM retinoic acid (Sigma-Aldrich), and 5 μM Y27632 (Millipore Sigma) for 24 hours. The medium was next replaced with Advanced DMEM/Glutamax supplemented with 5 μM CHIR99021 and 2 μM retinoic acid for 2 days. Cells were subsequently passaged at a 1:6 ratio in a medium supplemented with 2 μM SB431542 (Sigma-Aldrich) and cultured until they reached confluency. Finally, cells were maintained in fibroblast growth medium (Promocell) supplemented with 20 ng/mL bFGF (Thermo Fisher Scientific) and 10 μM SB431542. The purity of the cardiac fibroblasts was assessed using phycoerythrin-conjugated human CD90 antibody (12-0909-42, Thermo Fisher Scientific), human DDR2 Alexa-488-conjugated antibody (FAB25381G, R&D Systems), and anti-human CD140a (Platelet-derived growth factor receptor α, PDGFRα) antibody (323511, Biolegend), analyzed using a MACSQuant Analyzer 10 flow cytometer (Miltenyi Biotec). An overview of the workflow from patient recruitment to iPSC differentiation and EV treatment is illustrated in Figure 1.

### Patient recruitment, primary atrial fibroblast isolation and culture

Human atrial fibroblasts were isolated from atrial appendages collected during cardiac surgery after informed consent using a protocol approved by the University of Ottawa Heart Institute Research Ethics Board. Informed consent was obtained from all participants. As previously described, atrial tissue was digested in a mixture of collagenase IV and 2.5% trypsin (Thermo Fisher Scientific) 29-31. Cells were then cultured in Dulbecco's Modified Eagle High Glucose medium supplemented with 10% fetal bovine serum, 1% L-glutamine and 1% penicillin-streptomycin (Thermo Fisher Scientific).

### Human heart-derived cell culture and EV isolation

Human heart-derived cells were isolated from atrial appendages collected during cardiac surgery after informed consent using a protocol approved by the University of Ottawa Heart Institute Research Ethics Board 8, 32. Informed consent was obtained from all participants. Cell products were manufactured to clinical-grade release standards in Biospherix units at The Ottawa Hospital Cell Manufacturing Facility. Human heart-derived cells were cultured in NutriStem XF media at 5% oxygen conditions. Conditioned media were generated over a 48-hour culture period at 1% oxygen conditions, using Iscove's Modified Dulbecco's Medium supplemented with 1% exosome-depleted fetal bovine serum (Thermo Fisher Scientific). EVs were isolated using differential ultracentrifugation, where conditioned media were cleared first at 10,000 xg for 30 min, followed by EV isolation/ pelleting at 100,000 xg for 180 mins. EV characterization was conducted via immunoblotting for CD63 (10628D, Thermo Fisher Scientific), CD81 (10630D, Thermo Fisher Scientific), and in the absence of the cellular marker Calnexin (sc-23954, Santa Cruz Biotechnology). EV concentration was determined using nanoparticle tracking analysis with a Nanosight LM10 (Malvern instruments). EV dosing was normalized to particle counts determined by nanoparticle tracking analysis. ProcartaPlex™ Human Exosome Characterization Panel, 6plex (EPX060-15845-901, Thermo Fisher Scientific), was utilized for EV marker characterization as per manufacturer's protocol.

### RNA isolation and transcriptional profiling

Cells were seeded at a concentration of 100,000 cells per well in a 6-well plate and incubated for 24 hours. After this period, the cells were treated either with 50,000 EV particles per cell or with phosphate-buffered saline. Twenty-four hours post-treatment, cells were lysed in Qiazol, and RNA was extracted using the miRNeasy Micro Kit (Qiagen).

Reverse transcription was conducted on 200 ng of the isolated RNA (iScript DNA Synthesis Kit, Bio-Rad), followed by a reverse transcription-quantitative polymerase chain reaction (SsoAdvanced Universal SYBR Green Supermix, Bio-Rad) according to the manufacturer protocol. Expression fold changes relative to control samples were calculated using the 2-ΔΔCt method, with 18S RNA levels serving as the normalization control for mRNA quantification. Target genes included NLRP3 (*Hs.PT.58.39303321*, Integrated DNA Technologies), IL-18 (*Hs.PT.58.25675872*, Integrated DNA Technologies), and IL-1β (*Hs.PT.58.1518186*, Integrated DNA Technologies).

For miRNA isolation and quantification, RNA was initially extracted from either 100,000 heart-derived cells or 3x10^10^ EV particles using the miRNeasy Kit (Qiagen) as per the manufacturer instructions. Quantification of miR-23a-3p and miR-100 was performed using the miRCURY RT Kit (miRCURY LNA SYBR Green PCR kit, Qiagen), and miRCURY miRNA Assay Primers (Qiagen). Fold changes in miRNA expression were calculated using the 2-ΔΔCt method, with U6 levels serving as the normalization control.

### EV uptake by primary and iPSC-derived atrial fibroblasts

Heart-derived cells were labeled using 1,1'-dioctadecyl-3,3,3',3'-tetramethylindodicarbocyanine, 4-chlorobenzenesulfonate (DiD; V22887, Thermo Fisher Scientific) according to the manufacturer protocol prior to EV isolation from conditioned media, as outlined above. These DiD-labeled EVs were used to track their uptake in atrial fibroblasts and iPSC-derived atrial fibroblasts. Briefly, cells were seeded in a 6-well plate and incubated for 24 hours prior to treatment with 100,000 EVs/ cell for 24 h. After these treatments, flow cytometry analysis was conducted to evaluate EV uptake using the MACSQuant Analyzer 10 Flow Cytometer (Miltenyi Biotec).

### Impact of inflammation and EVs on primary and iPSC-derived atrial fibroblast proliferation

Manual cell counts were conducted after treating cells with interleukin 6 (IL-6) and Transforming growth factor beta 1 (TGF-β1) at concentrations of 10 ng/mL (Cell Signaling). Cells were either cultured with or without EVs at a concentration of 50,000 EVs/cell. Cells without EV or cytokine treatments served as the baseline. For cytokine-stimulation proliferation experiments, EVs were added concurrently with IL-6 or TGF-β1 (time 0) and maintained for the duration of the assay, modeling attenuation of fibro-inflammatory signaling at the time of exposure.

To explore whether EVs could attenuate an established proliferative phenotype, fibroblasts were first exposed to inflammatory stimulation for a defined 24 hour period prior to EV administration for another 24 hours. EVs (or vehicle) were then added without removal of the inflammatory stimulus, and proliferation was assessed over the subsequent incubation period as described above.

### NLRP3 inflammasome activation in primary and iPSC-derived atrial fibroblasts

NLRP3 inflammasome and downstream mediators of NLRP3 activation were evaluated in both human fibroblasts and iPSC-differentiated atrial fibroblasts. To model attenuation of inflammasome priming and activation, cells were pre-treated with EVs prior to exposure to LPS and nigericin. 100,000 cells/ well were seeded in a 12-well plate for 24 hours followed by treatment with 50,000 EVs/ cell for 24 hours before exposure to 100 ng/mL lipopolysaccharide (LPS, Sigma) for 4 hours followed by 20 µM nigericin (Sigma) 1 hour to activate the pathway. Supernatants were then collected and assayed using ELISAs for Caspase-1 (R&D systems).

### Statistical analysis

All statistical methodologies and graphical representations of the data are specified in the figure legends corresponding to each data panel, using GraphPad Prism version 10.4. Data are presented as mean ± standard deviation (SD). For experiments involving comparisons across multiple groups, an ordinary one-way ANOVA was performed, followed by Dunnett's post-hoc test when comparing treatment groups to a single control. For comparisons involving only two groups, an unpaired Student's t-test was used. Bartlett's test was employed to assess the homogeneity of variances prior to conducting parametric tests. Categorical variables were analyzed using Fisher's exact test. A P-value of ≤0.05 was considered statistically significant. All experiments were performed in triplicate unless otherwise specified.

## Supplementary Material

Supplementary figures.

## Figures and Tables

**Table 1:** Clinical and demographic characteristics of patients contributing induced pluripotent stem cell (iPSC) and primary atrial fibroblast samples.

Variable	All patients (n = 18)	Low risk (n = 9)	High risk (n = 9)	P value (low vs. high)	Primary atrial fibroblasts	P value (primary cells vs. iPSC donors)
Age (years)	64 ± 12	57 ± 10	74 ± 5	<0.01	63 ± 7	0.88
Sex (% female)	28%	22%	33%	NS	100%	0.04
Body mass index (kg/m²)	31 ± 7	32 ± 7	29 ± 6	NS	31 ± 7	0.69
Serum creatinine (μmol/L)	94 ± 16	90 ± 11	98 ± 20	NS	62 ± 6	0.02
eGFR (mL/min/1.73 m²)	90 ± 31	109 ± 17	68 ± 29	<0.01	90 ± 31	-
Postoperative AF score	1.1 ± 1.3	0	2.4 ± 0.7	<0.01	-	-
Age component	0.9 ± 1.1	0	1.9 ± 0.6	<0.01	-	-
Valve surgery component	0.3 ± 0.5	0	0.6 ± 0.5	0.015	-	-
Documented postoperative AF (%)	33%	22%	44%	0.69	-	-
AF onset after surgery (days)	3.7 ± 1.2	3.5 ± 0.7	3.8 ± 1.5	0.84	-	-

AF, atrial fibrillation; eGFR, estimated glomerular filtration rate; iPSC, induced pluripotent stem cell. Data are presented as mean ± standard deviation or %. Continuous variables were compared using the Student t-test; categorical variables using Fisher's exact test.

## Data Availability

All data generated or analyzed during this study are included in this published article and its Supplementary Information files. Additional information is available from the corresponding author upon reasonable request.

## Abbreviations

AF: Atrial fibrillation
DiD: 1,1'-dioctadecyl-3,3,3',3'-tetramethylindodicarbocyanine, 4-chlorobenzenesulfonate
EV: Extracellular vesicles
IL: Interleukin
iPSC: Induced pluripotent stem cell
LPS: Lipopolysaccharide
miR: MicroRNA
NLRP3: Nucleotide-binding domain, leucine-rich repeat family, pyrin domain containing 3
pAFB: Primary atrial fibroblast
PDGFRα: Platelet-derived growth factor receptor α
STAT3: Signal Transducer and Activator of Transcription 3
TGF-β1: Transforming Growth Factor Beta 1
