# Supplementary material for: Human atrial extracellular vesicles suppress NLRP3 inflammasome activation and profibrotic signaling in a patient-specific iPSC model of postoperative atrial fibrillation
Source: Theranostics. 2026 Mar 30;16(11):5804–15. doi: 10.7150/thno.127433 (PMC13141743; doi:10.7150/thno.127433)
Supplement: Supplementary file 1 — Supplementary figures. [file thnov16p5804s1.pdf]

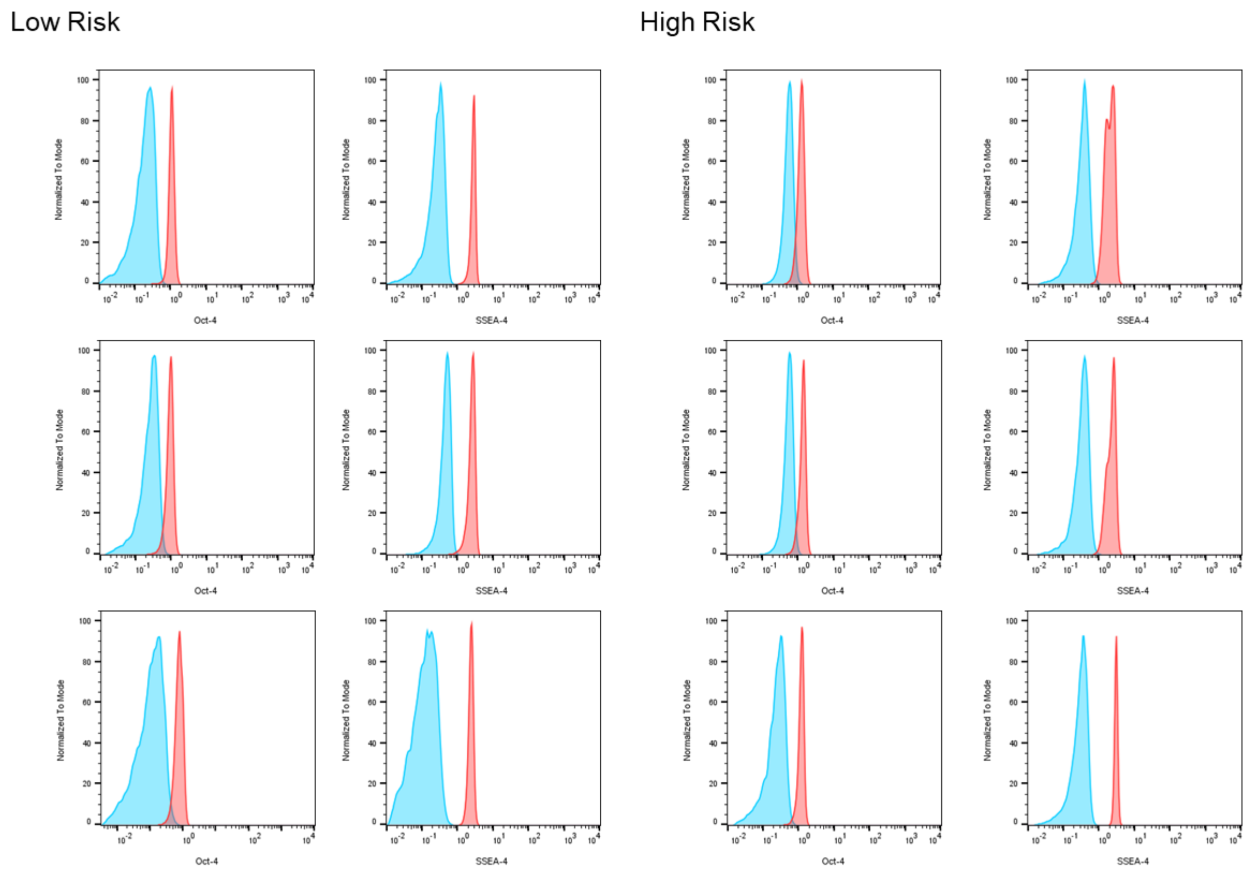

Supplemental Figure 1 Representative quantified flow cytometry showing robust expression of Oct4 and SSEA4 in reprogrammed iPSCs at passage 30.

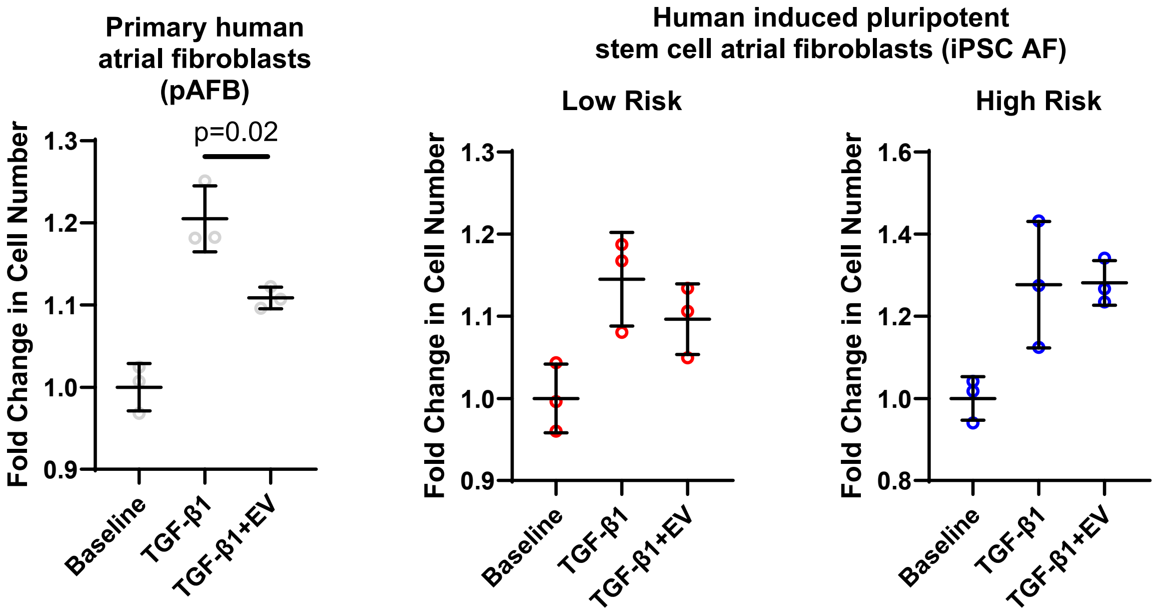

Supplemental Figure 2 Delayed extracellular vesicle (EV) administration in human atrial fibroblast models of postoperative atrial fibrillation (AF). Fold change in cell number in primary human atrial fibroblasts (pAFB, grey) following initial exposure to transforming growth factor–β1 (TGF-β1) to induce proliferation, with EVs administered subsequently in a delayed-treatment paradigm. Delayed EV administration significantly attenuated TGF-β1–induced proliferation ($p = 0.02$). Fold change in cell number in atrial fibroblasts derived from human induced pluripotent stem cells from patients at low risk of postoperative AF (LR iPSC-AF, red) and high risk of postoperative AF (HR iPSC-AF, blue) subjected to the same delayed-treatment paradigm. In contrast to pAFB, delayed EV treatment did not significantly reduce established proliferation in LR or HR iPSC-derived fibroblasts. Data are presented as mean ± SD; $n = 3$ biological replicates per group. Statistical comparisons were performed using unpaired Student's t-tests; $p < 0.05$ was considered significant.

Three independent EV samples from three different donors (i.e., 3 biological replicates)

CD63

EV sample 1 (Biological Sample 1) | EV sample 2 (Biological Sample 2) | EV sample 3 (Biological Sample 3)

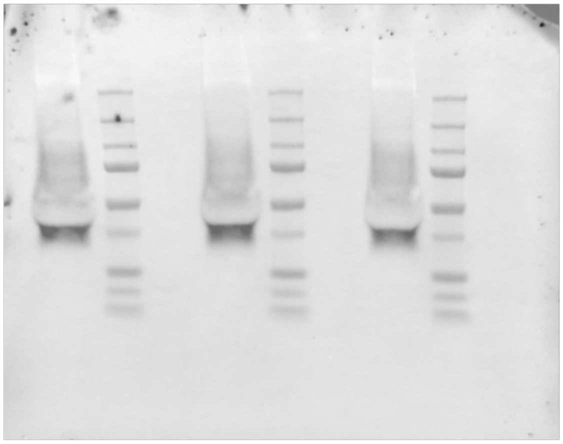

CD81

EV sample 1 (Biological Sample 1) | EV sample 2 (Biological Sample 2) | EV sample 3 (Biological Sample 3)

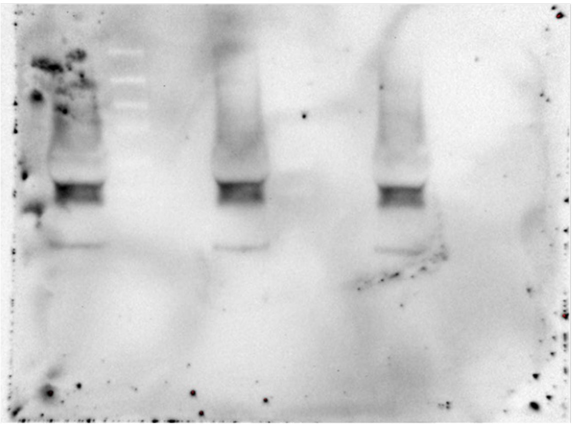

Calnexin

EV sample 1 (Biological Sample 1) | EV sample 2 (Biological Sample 2) | EV sample 3 (Biological Sample 3)

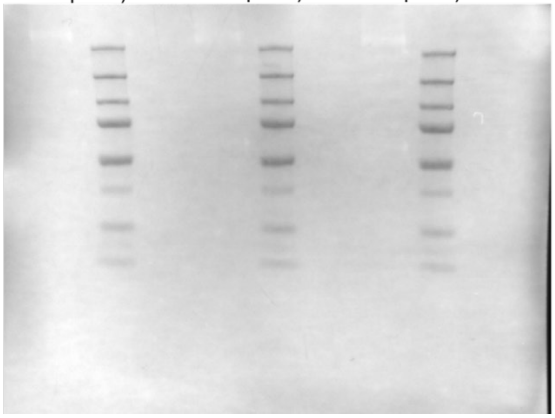

Supplementary Figure 3 Uncropped Western blot images used for extracellular vesicle characterization. Complete, unprocessed original blot images corresponding to the data presented in the manuscript are shown. Blots were probed for the extracellular vesicle markers CD63 and CD81 and for calnexin as a negative control for cellular contamination. Each lane represents an independent biological EV sample derived from separate preparations (n = 3 biological samples) that were run on the same membrane.
